# Reduced APOBEC3H Variant Anti-Viral Activities Are Associated with Altered RNA Binding Activities

**DOI:** 10.1371/journal.pone.0038771

**Published:** 2012-07-30

**Authors:** Anjie Zhen, Juan Du, Xiaohong Zhou, Yong Xiong, Xiao-Fang Yu

**Affiliations:** 1 Department of Molecular Microbiology and Immunology, Johns Hopkins Bloomberg School of Public Health, Baltimore, Maryland, United States of America; 2 First Affiliated Hospital, Jilin University, Jilin, China; 3 Department of Molecular Biophysics and Biochemistry, Yale University, New Haven, Connecticut, United States of America; Institut Pasteur, France

## Abstract

APOBEC3H (A3H) is a member of the APOBEC3 family of proteins with varying activities against retroviruses and retrotransposons. The A3H gene contains several single nucleotide polymorphisms and up to seven haplotypes have been detected in humans. Although variations in anti-viral function among A3H haplotypes are not fully understood, only 15N105R-containing A3H variants are known to have potent activities against Vif-deficient HIV-1. Unique motif RLYY(F/Y)W of APOBEC3G (A3G) and APOBEC3F (A3F) required for 7SL RNA binding and HIV-1 incorporation is also conserved in all A3H variants. Like A3G, A3H HapII also demonstrated high binding affinity to host small RNAs such as 7SL and Y RNAs. Mutation of a critical amino acid, W115A resulted in reduced expression level, decreased affinity for 7SL RNA, impairment of virion packaging and reduced anti-viral activity. By comparison, A3H HapI had lower binding affinities to host small RNAs and reduced efficiency of virion incorporation, resulting in significantly reduced anti-viral activity. The SNP ΔN15 commonly found in A3H HapIII and HapIV abolished their abilities to associate with RNAs, and A3H HapIIΔ15N failed to package into HIV-1 virions or exhibited any anti-viral activity. Finally, we showed that A3H variants had distinct cellular localization patterns, which correlated with their different RNA binding affinities. Thus, Pol-III RNA such as 7SL RNA binding is a conserved feature of potent anti-HIV human APOBEC3 cytidine deaminases.

## Introduction

The apoliprotein B mRNA-editing catalytic polypeptide 3 (APOBEC3) protein family consists of seven family members (APOBEC3A, -B, -C, -DE, -F, -G, and –H) with diverse anti-viral and anti-retrotransposition activities. Select family members can potently suppress retrovirus replication by editing the viral genome via cytidine deamination and other mechanisms [1], [2], [3], [4], [5], [6], [7]. In order to successfully replicate, HIV-1 encodes Vif, a SOCS box containing protein which hijacks the host E3 Cul5 ubiquitin ligase system. This viral-host complex induces polyubiquitination and degradation of several APOBEC3 molecules [3], [7], [8], [9], [10], [11], [12], [13], [14].

APOBEC3H (A3H) is the only APOBEC3 protein that contains a single copy of the Z3 type APOBEC3 catalytic domain. This domain is conserved in mammalians and has been positively selected during primate evolution [15], [16]. A3H transcripts have been detected in various human tissues including peripheral blood monoclear cells, liver, and skin among other tissues. Its expression can also be induced by IFN-α in HIV-1 target cells [16], [17], [18], [19]. A number of SNPs were reported for the human A3H gene in the Single Nucleotide Polymorphism (SNP) database at the National Center for Biotechnology Information (NCBI, www.nicbi.nlm.nih.gov/projects/SNP). Four of the non-synonymous SNPs (R18L, G105R, K121E/D, E178D) and one codon deletion (Δ15N) in A3H have been characterized in previous reports [14], [16], [17], [18], [20], [21], [22], [23], [24]. Initially, four A3H haplotypes were identified to be circulating in the human population [16], [17], [22], namely HapI (18R/105G/121K/178E), HapII (18R/105R/121E/D/178D), HapIII (d15N/18R/105R/121E/D/178D) and HapIV (d15N/18L/105R/121E/D/178D). Recently, additional haplotypes were discovered that have different combinations of the SNPs: HapV (18R/105R/121D/178E), HapVI (d15N/18L/105G/121K/178D) and HapVII (18R/105R/121K/178E) [24].

Different A3H haplotypes have diverse anti-viral activity. Amino acids 105R and 15N determine protein stability, and only A3H containing15N105R has strong anti-viral activity against ΔVif HIV-1 [14], [16], [17], [18], [22], [23], [24]. Whether A3H can be efficiently degraded by Vif is still controversial, but two studies have shown that Vif can induce degradation of A3H HapII, but not HapI [17], [20]. In addition, a single amino acid, 121 K/D/E, determines A3H interaction with Vif and Vif-induced degradation [7], [14]. LaRue *et* *al.* also reported that lentiviral Vif evolved to specifically degrade A3Z3-type proteins (e.g., A3H) of its own mammalian host [10], arguing for the importance of Z3 proteins in an organism's fight against lentivirus infection.

**Figure 1:**
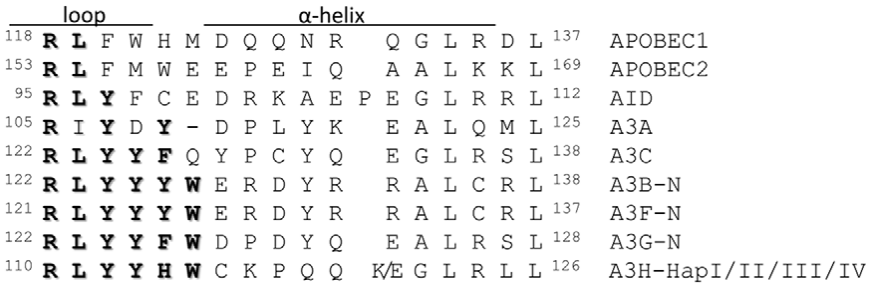
Conservation of the unique RNA binding motif RLYY(F/Y/H)W in A3H variants. Amino acid sequences from various human AID/APOBEC proteins were aligned using ClustalW2. A unique motif RLYY(F/Y/H)W on the N-terminus of A3G and A3F required for their binding of 7SL RNA and virion incorporation is conserved on all A3H variants.

However, the underlying mechanism of diverse anti-viral activities of A3H variants is still not clear. As for A3G and A3F, its anti-viral effects require efficient packaging into the HIV-1 virion [5], [25], . In the absence of Vif, A3G and A3F virion incorporation requires the RNA binding nucleo-capsid (NC) domain of Gag and many studies have found that RNA is required for the interaction between Gag and A3G [25], [26], [28], [29], [30]. Some studies have reported viral genomic RNA is required for efficient A3G packaging [31], [32], while others argued that cellular RNA, especially 7SL RNA is responsible for mediating A3G and A3F packaging [27], [33]. Host 7SL RNA is most abundant in HIV-1 particle and appeared to be specifically enriched in HIV-1 virion [34]. A3G and A3F have high affinity with 7SL RNA and association with 7SL RNA is crucial for anti-viral activity as well as accurate viral core targeting of various APOBEC3 proteins [35].

**Figure 2:**
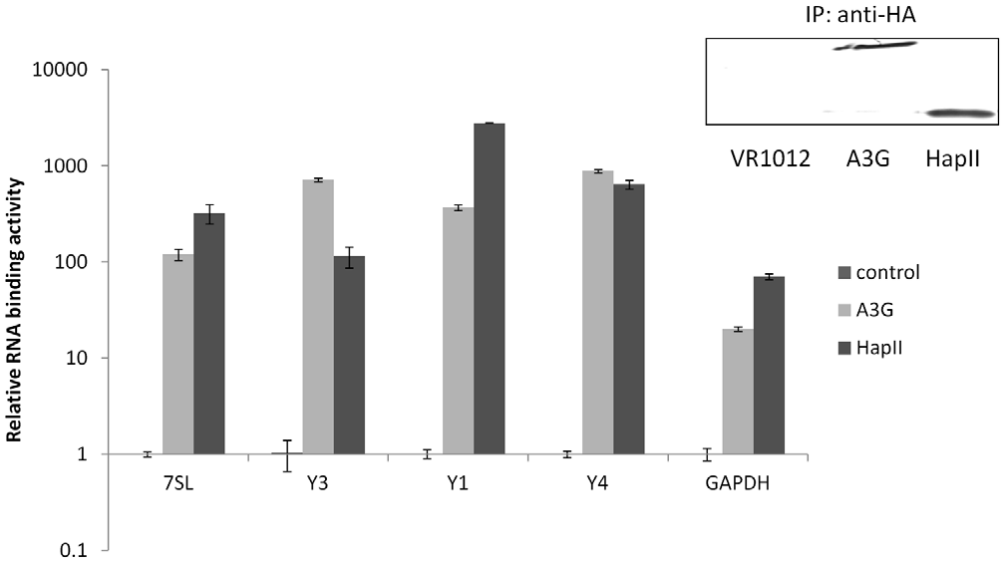
RNA binding activities of A3G and A3H HapII. To compare the RNA binding affinities of A3H HapII and A3G, 293T cells were transfected with empty vector, HA-tagged A3H HapII or A3G. Forty-eight hours post-transfection, the cell lysates were collected and immunoprecipitated using anti-HA affinity matrix. The immunoprecipitated RNAs were purified and reverse transcribed. The relative amounts of 7SL, Y RNAs and GAPDH RNA associated with A3G and A3H HapII in comparison to the control were measured by qRT-PCR.

Previous studies have reported that A3H HapII incorporation into HIV-1 requires the Gag NC domain; however, NC is not required for A3H HapI incorporation into virions [23]. Wang, et al [36] showed little virion packaging for HapI, III, IV, and VI. However, these haplotypes were poorly expressed in the cell and it is unknown if these haplotypes have virion packaging ability when expressed to a similar level as A3H HapII. In addition, it is not completely clear how A3H is packaged into HIV-1. Here we report that like A3G, A3H anti-viral activity and virion incorporation are also dependent on RNA binding activity, and that the differences in RNA binding activity determine anti-viral potency and cellular localization.

**Figure 3:**
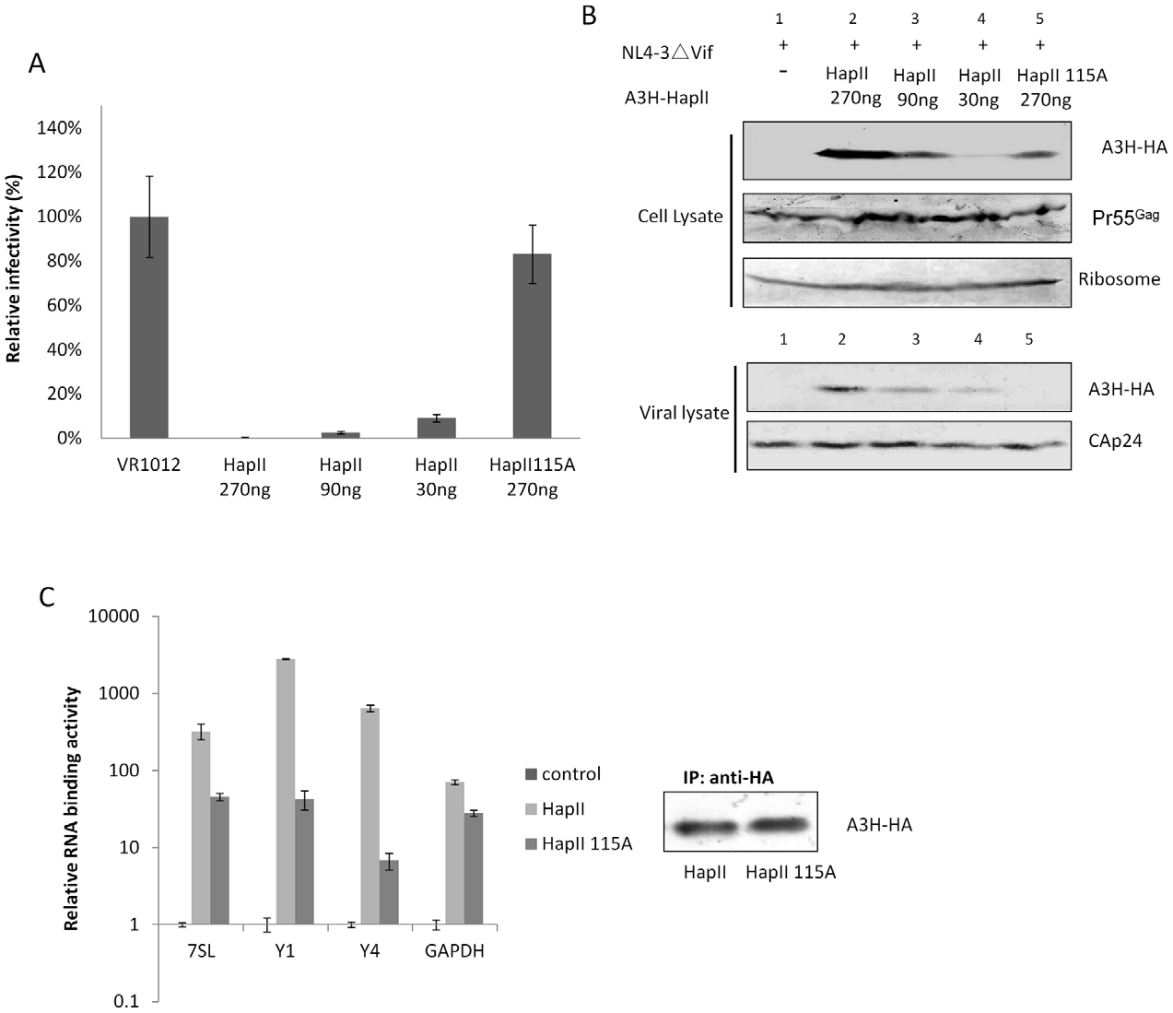
Single point mutation on 115W impairs A3H HapII RNA binding activity and viral packaging. A) A3H HapII115A showed no anti-viral activity against HIV-1 NL4-3ΔVif compared to A3H HapII. B) A3H HapII115A had reduced protein expression level compared to A3H HapII (compare cell lysates in lanes 2 and 5), but even when expressed at similar levels (compare cell lysates in lanes 3 and 5), HapII 115A lost its ability to be packaged into the virus (viral lysate lane 5). C) A3H HapII115A had impaired ability to bind to 7SL, Y RNAs as well as GAPDH RNA.

## Materials and Methods

### Plasmids

Infectious molecular clones of wild-type (WT) HIV-1, pNL4-3 and pNL4-3Δvif, were obtained from the National Institutes of Health AIDS Research and Reference Reagent Program (NIH-ARRRP), Division of AIDS, National Institute of Allergy and Infectious Diseases. pCMV-A3H-HapI-V5 was a kind gift of Yonghui Zheng, Michigan State University. pCMV-A3H-HapII-V5 and A3H-HapI-105R-V5, A3H-HapI-105R121D-V5, A3H-HapI-121D-V5 was constructed by point mutation as described previously [20]. The A3H variant sequences (A3H-HapI-HA, A3H-HapI-105R-HA, A3H-HapI105R121D-HA, A3H-HapII-HA) were cloned into the VR1012 vector for enhanced expression in mammalian cells after amplification of the genes from the respective pCMV-V5 plasmids using *Pfu* polymerase (Stratagene) and the following primers: BgIII-HA-hA3H-3: 5′-GTACAGATCTTCAAGCATAATCCTGGAACATCGTATGGATAGGACTGCTTTATC-3′; SaII hA3H_5: 5′-GTACGTCGACATGGCTCTGCTGACAGCCG-3′.

**Figure 4:**
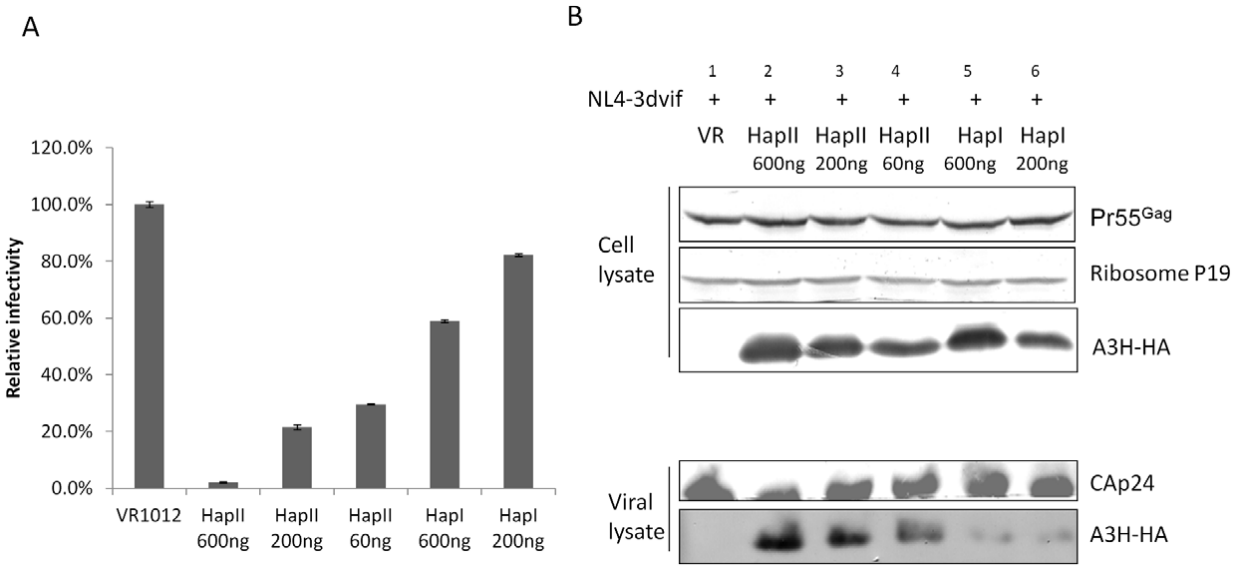
A3H HapI showed an impaired ability to package into HIV-1 virions. To compare anti-viral activity and packaging of A3H HapI and HapII, pNL4-3Δvif was transfected with decreasing doses of A3H HapII and HapI. A) A3H HapI showed significantly reduced anti-viral activity compared to A3H HapII. B) Even when A3H HapI was expressed at a similar level as A3H HapII (compare cell lysates in lanes 3 and 5), it had a reduced ability to be packaged into the virion (compare viral lysates in lanes 3 and 5).

### Cell culture, transfections, viral infectivity (MAGI) assays and antibodies

293T cells were obtained from ATCC (CRL-11268™) and MAGI-CCR5 cells were obtained from AIDS Research and Reference Reagents Program (Catalogue no. 3522). Both cell lines were maintained in Dulbecco's modified Eagle's medium (Invitrogen, Carlsbad, CA) with 10% fetal bovine serum and 25 µg/ml gentamicin (Sigma, St. Louis, MO) and transfected or infected as previously described [13]. Transfections were performed with Lipofectamine 2000 (Invitrogen) as instructed by the manufacturer. The viral infectivity (MAGI) assay was performed as previously described [13]. In brief, viruses were produced by transfecting 293T cells in a 25 cm2 flask with 3 μg of pNL4–3 or pNL4–3Δvif and with different doses of A3H expression vector or an empty vector as a control. The viruses were harvested from the supernatant, and the cells were reserved for immunoblotting to monitor A3H degradation. Infectivity was assessed at 48 h post-infection and normalized to the input CAp24 of the virus detected by anti-p24 antibody (NIH-ARRRP). The following antibodies were obtained from commercial sources: anti-HA monoclonal antibody (MAb) (Covance, MMS-101R-10000), anti-myc MAb (Sigma, M5546), and anti-actin MAb (Sigma, A3853). The anti-Vif antibody was obtained from the NIH-ARRRP (#2221). The mouse anti-V5 antibody was from Invitrogen (R96025), and the rabbit anti-GFP antibody was from Abcam (ab6556-25).

**Figure 5:**
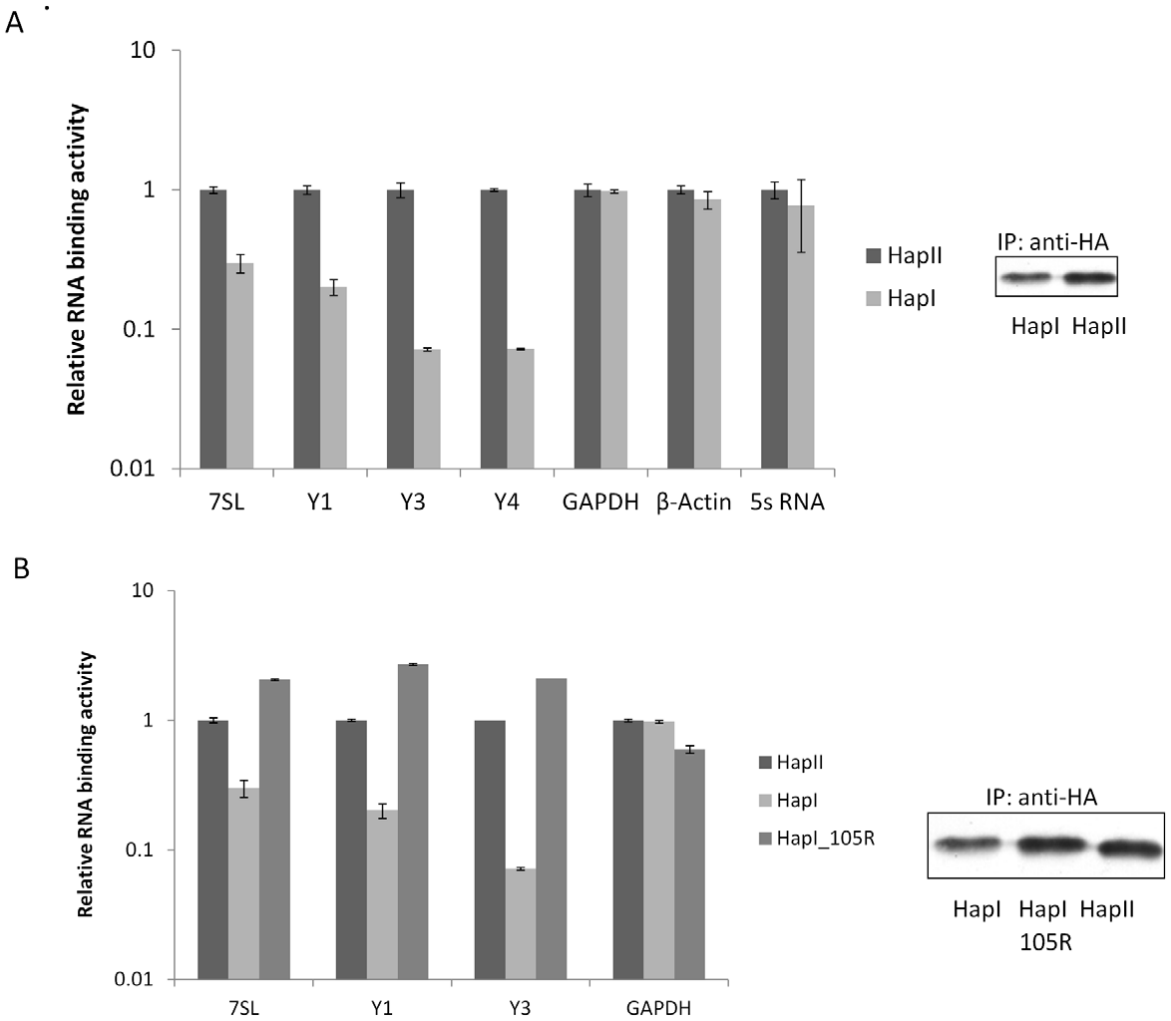
A3H HapI had a reduced ability to associate with 7SL and Y RNAs. Single mutation G105R restored its RNA binding activity. A) To compare RNA binding activities between A3H HapII and HapI, 293T cells were transfected with HA-tagged A3H HapII and HapI, and the cell lysates were immunoprecipitated using anti-HA affinity matrix agarose beads. Immunoprecipitated RNA were purified and reverse transcribed, and the relative amounts of 7SL, Y RNAs, GAPDH, β-actin and 5s RNA were measured using qRT-PCR. B) Relative RNA binding activities of A3H HapI and HapI105R compared to HapII.

### Immunoprecipitation and immunoblot analysis

293T cells were harvested 48 h after transfection, washed with PBS and lysed in lysis buffer (50 mM Tris, pH 7.5, with 150 mM NaCl, 1% Triton X-100 and Roche Complete Protease Inhibitor Cocktail Tablets) at 4°C for 30 min, followed by centrifugation at 10,000×*g* for 30 min. For the immunoprecipitation experiment comparing RNA binding activities of A3H HapII and HapIIΔ15N, the transfected cell lysates were sonicated to ensure efficient lysis of both A3H HapII and HapIIΔ15N from the nucleus. The HA-tag immunoprecipitation was carried out by mixing lysates of transfected cells from a T25 flask with a 25-μl bead volume of anti-HA antibody-conjugated affinity matrix (Roche) and incubating the mixture at 4°C for 3 h. The samples were washed six times with wash buffer (20 mM Tris, pH 7.5, with 100 mM NaCl, 0.1 mM EDTA and 0.05% Tween-20). The beads were then eluted with protein 2× loading buffer. The eluted materials were then analyzed by SDS-PAGE and immunoblotting with the appropriate antibodies.

**Figure 6:**
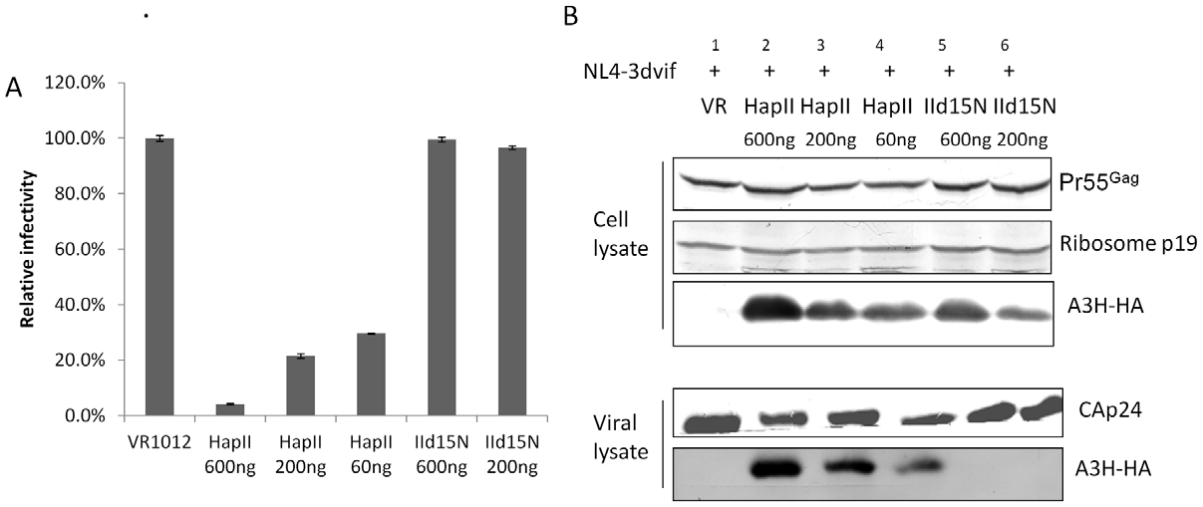
The Δ15N deletion abolished A3H HapII anti-viral activity and virion incorporation. To compare anti-viral activity and packaging of A3H HapII and HapIIΔ15N, pNL4–3Δvif was transfected with decreasing doses of A3H HapII and HapIIΔ15N. A) A3H HapIIΔ15N had no anti-viral activity compared to A3H HapII. B) Even when expressed at similar levels as A3H HapII (cell lysates in lane 3 and 5), A3H HapIIΔ15N completely lost its ability to be packaged into HIV-1.

### Quantitative real-time PCR (qRT-PCR)

RNA obtained from immunoprecipitated samples were treated with DNase I by incubation in 10 µl of diethyl pyrocarbonate (DEPC)-treated water with 1x RQ1 RNase-Free DNase buffer, l µl of RQ1 RNase-free DNase (Promega) and 4 U of RNase inhibitor (New England Biolabs) for 30 min at 37°C. The DNase was inactivated by the addition of 1 µl RQ1 DNase stop solution and incubated at 65°C for 10 min. The RNA was reverse transcribed by using random primers and the Multiscribe reverse transcriptase from the High-Capacity cDNA Archive Kit (Applied Biosystems) according to the manufacturer's instructions. The cDNA was either undiluted or serially diluted in DEPC-treated water before input into the real-time reaction to ensure that the amplification was within the linear range of detection.

**Figure 7:**
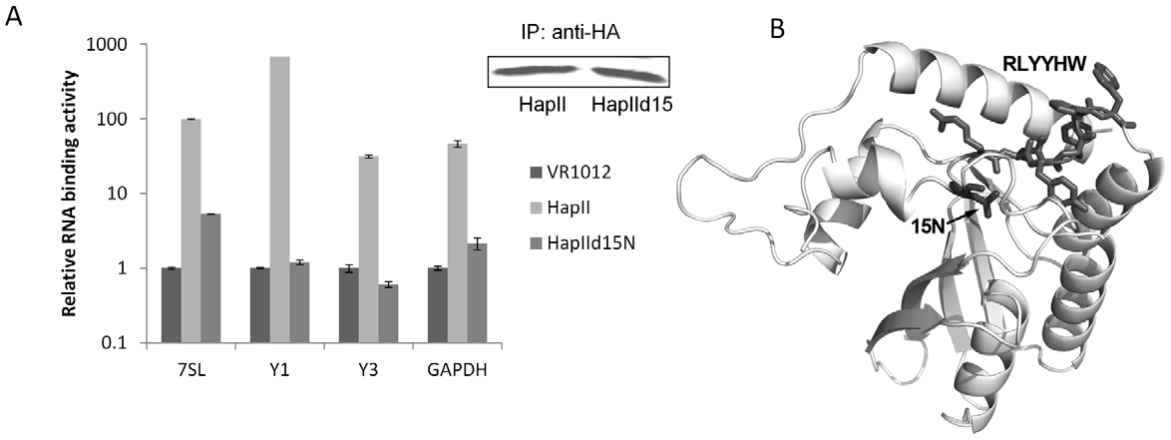
A3H HapIIΔ15N has little RNA binding affinity. A) To compare the RNA binding activity between A3H HapII and HapI, 293T cells were transfected with HA-tagged A3H HapII and HapI, and the cell lystes were immunoprecipitated using anti-HA affinity matrix. Immunoprecipitated RNA were purified and reverse transcribed, and the relative amounts of 7SL, Y RNAs and GAPDH were measured. B) Homology modeling of A3H HapII showing that 15N lies close to the putative RLYYHW RNA binding motif.

The ABI 7000 sequence detection system (Applied Biosystems, Carlsbad, CA) was used for the qRT-PCR amplifications. All primers were synthesized by Invitrogen, and fluorescence-tagged probes were synthesized by Applied Biosystems. Agarose gel analysis was used to verify that each primer pair produced single amplicons, and the identities of the PCR products were verified by cloning and sequencing. qRT-PCR was performed by using SYBR Green. Each 20-µl reaction mixture contained 1 µl forward and reverse specific primers (10 µM each), 10 µl 2x SYBR Green PCR Master Mix, 5 µl RNase-free water and 3 µl template cDNA. The reactions were performed under the following conditions: 50°C for 2 min and 95°C for 10 min, followed by 40 cycles of 95°C for 15 s and 60°C for 1 min, followed by a dissociation protocol. Single peaks in the melting curve analysis indicated specific amplicons. The target sequences were amplified using the following primer pairs: 7SL RNA, forward (5′-ATCGGGTGTCCGCACTAAG-3′), reverse (5′-CACCCCTCCTTAGGCAACCT-3′), Y1 RNA, forward (5′-GGCTGGTCCGAAGGTAGTGA-3′), reverse (5′-AAAAGACTAGTCAAGTGCAGT-3′); Y3 RNA, forward (5′-GGCTGGTCCGAGTGCAGT-3′), reverse (5′-AAAAGGCTAGTCAAGTGAAGC-3′); Y4 RNA, forward (5′-GGCTGGTCCGATGGTAGTG-3′), and reverse (5′-AAGCCAGTCAAATTTAGCAGTGGG-3′); Y5 RNA, forward (5′-AGTTGGTCCGAGTGTTGTGGGT-3′), and reverse (5′-ACAGCAAGCTAGTCAAGCGCG-3′); GAPDH, forward (5′-GCAAATTCCATGGCACCGT-3′), and reverse (5′-TCGCCCCACTTGATTTTGG-3′); and 5S RNA, forward (5′-TTCAGCGTCTACGGCCATAC-3′), and reverse (5′-AGCCAAAGAAAAAGCCTAC-3′);

**Figure 8:**
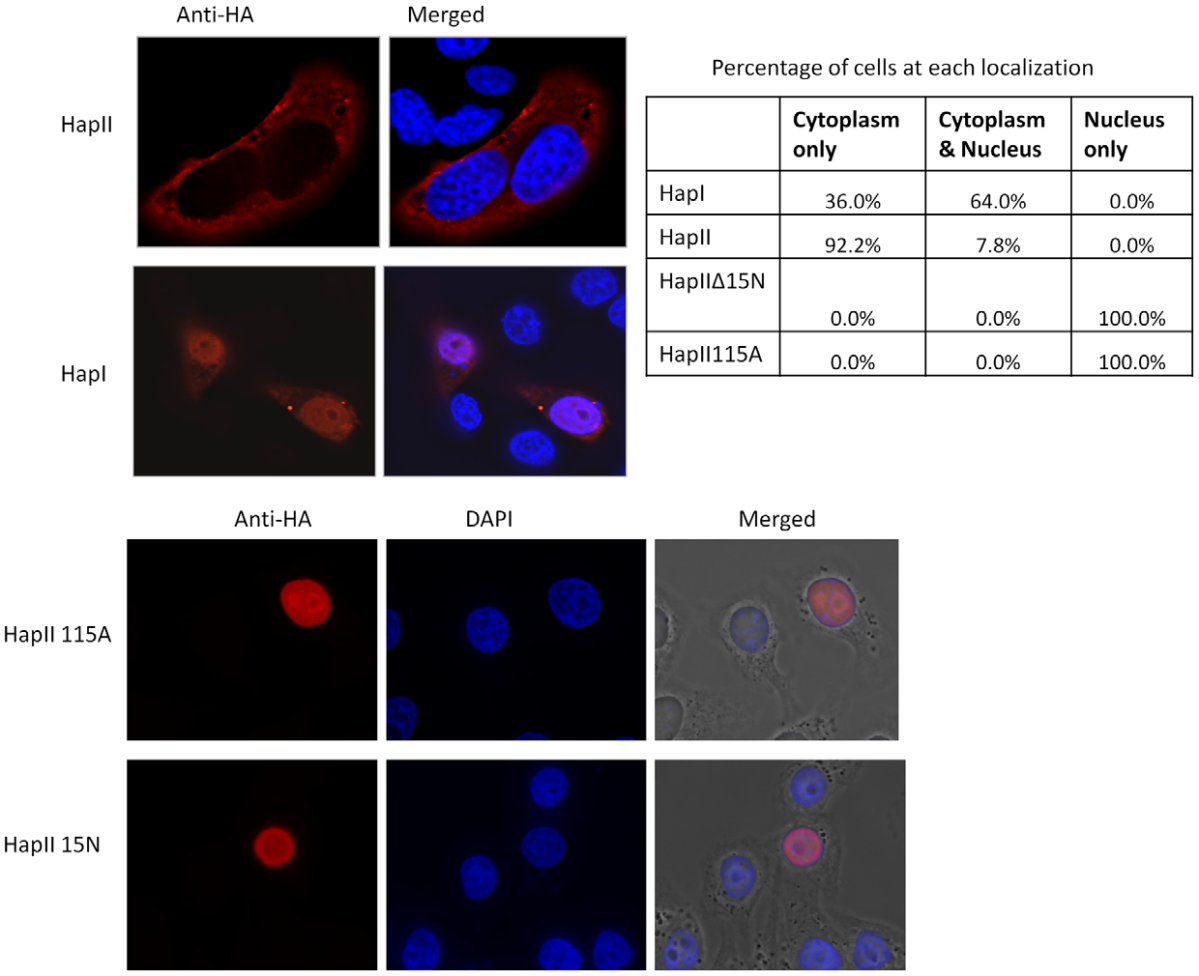
Cellular localization of A3H variants. Hela cells were transfected with various A3H variant constructs, fixed, permeabilized and stained with a mouse anti-HA antibody followed by a secondary Texas Red conjugated anti-mouse antibody. The nucleus was stained with DAPI. The cells were examined by deconvolution microscopy as described in Methods. A series of Z-stack images were acquired with z–step of 0.2 µm and raw image sequences were processed with velocity imaging system and deconvolved. Various cellular localization patterns of the A3H variants were observed. The numbers of cells observed in each location were counted and summarized as percentages of total cells in the table.

### Immunofluorescent staining

Hela cells were transfected with various A3H expression plasmids and were passed onto cover slips 24 h after transfection and allowed to reattach to the cover slip for another 24 h. The cells are then fixed with 4% paraformaldehyde, permeabilized with 0.5% Triton and blocked with 10% BSA. HA-tagged A3H variants were stained with a mouse anti-HA antibody (Sigma, H9658) followed by a Texas Red conjugated anti-mouse antibody. The cell nucleus was stained with DAPI and examined by deconvolution microscopy using a Nikon i90 microscope. A series of Z-stack images were acquired with z–step of 0.2 µm, and raw image sequences were processed with a velocity imaging system and deconvolved.

### Modeling

Homology modeling of A3H-HapII was carried out using both the program Modeller [37] and the modeling server I-TASSER [38], which has been ranked as the best server in the recent CASP7 and CASP8 modeling contests [39]. Both Modeller and I-TASSER produced very similar models, suggesting convincing modeling results. Prior to modeling in Modeller, multiple sequence alignment was carried out for APOBEC2, the amino-terminal domain of A3G (A3G-NTD), carboxyl-terminal domain of A3G (A3G-CTD), and A3H-HapII by using the program MUSCLE [40]. The availability of crystal structures of two homologous APOBECs, APOBEC2 [41] and A3G CTD [42], [43], helped to validate and manually improve the alignment. The aligned sequences of A3G-NTD/A3H-HapII, APOBEC2, and A3G-CTD were input into Modeller for homology modeling using the two crystal structures as multiple templates, which improved the quality of the modeling. The high degree of homology among the proteins and the similarity of the resulting models impart confidence in the homology modeling, especially for the regions in which the conserved motif RLYYFW and A3H-HapII residues 15N, 105, 121, and 178 are located.

## Results

### A3H HapII binds host RNA with high affinity

Potent anti-HIV-1 cytidine deaminases possess unique RNA-binding abilities which contribute to HIV-1 packaging and anti-viral activity [35]. A3G and A3F both contain a conserved motif, RLYY(F/Y)W, demonstrated to be crucial for host RNA binding and HIV-1 virion incorporation [27], [35]. Among all known A3H variants, A3H HapII has the most potent anti-viral activity and can be packaged into virions efficiently [14], [22]. To determine the level of conservation of this RLYYF/YW motif, we aligned amino acid sequences from various human AID/APOBEC proteins. As shown in Figure 1, the RLYY(F/Y)W motif is conserved in A3B, A3G and A3F. Interestingly, the RLYYHW sequence was found in all A3H haplotypes.

A3G binds with high affinity to many cellular RNAs, notably 7SL RNA and Y RNAs [27], [44]. Indeed, binding to 7SL RNA has been proposed to be essential for A3G incapsidation in the HIV-1 viral core [27], [35], [45]. To test whether A3H HapII has similar RNA binding activity, 293T cells were transfected with either empty vector, A3G-HA or A3H HapII-HA constructs. The cell lysates were harvested 48 h post-transfection, and the A3 proteins were immunoprecipitated. RNAs bound to the immunoprecipitated A3 proteins were extracted and reverse transcribed. The levels of 7SL, Y1, Y3 and Y4 RNAs, as well as GAPDH RNA were then measured as fold increase compare to control using qRT-PCR. RNA binding activities of A3G and A3H HapII, relative to control, are presented in Figure 2. Similar to previous report [27], more than 100 fold 7SL RNA were co-precipitated with A3G-HA compared to control, while more than 300 fold of various Y RNA were associated with A3G-HA. A3H HapII demonstrated similar high binding affinities to 7SL, Y1, Y3 and Y4 RNAs. In particular, more than 300 folds of 7SL RNA were co-precipitated with A3H-HA compared to control.

### A3H HapII packaging in HIV-1 virions is dependent on host RNA binding

A previous report demonstrated that mutating A3G at amino acid 127 W to A within the RNA binding motif RLYYFW abolished its ability to bind 7SL and Y RNAs as well as reduced its antiviral activity against HIV-1 NL4-3ΔVif [45]. To determine if this motif, RLYYHW, is also important for A3H HapII RNA binding, we constructed a vector to express A3H HapII W115A and tested its RNA binding and anti-viral activities. 293T cells were co-transfected with pNL4-3Δvif and various amounts of A3H HapII or A3H HapII W115A expression plasmids. As shown in Figure 3A, A3H HapII demonstrated strong suppressive activity against the Δvif virus, while HapIIW115A lost the ability to suppress viral replication. A3H HapIIW115A was also expressed at lower levels compared to wild-type A3H HapII (Figure 3B, compare lanes 5 and 2). However, when expressed at equal levels (Figure 3B, lanes 3 and 5), A3H HapII W115A still could not suppress viral replication as efficiently as wild-type A3H HapII. In addition, A3H HapII W115A was barely detectable in the viral lysate (Figure 3B, lane 5), indicating that A3H HapII W115A lost its viral packaging ability. We next examined whether A3H HapII W115A may also have lost the ability to bind host RNAs. As shown in Figure 3C, compared to A3H HapII wild type, A3H HapIIW115A had significantly reduced RNA binding activities to Pol III RNAs such as 7SL, Y1 and Y4 RNAs. Interestingly, unlike A3G-W127A, which maintained its ability to associate with mRNAs such as GAPDH, A3H HapIIW115A also showed a reduced ability to associate with GAPDH mRNA, indicating that the RLYYHW motif plays a crucial role in general RNA binding and as a consequence, it is absolutely required for virion incorporation.

### A3H HapI has reduced ability to be packaged into HIV-1 virions and decreased anti-viral activity

Compared to A3H HapII, the A3H HapI protein is less stable and has weaker anti-viral activity [14], [16], [18], [22]. However, when expressed at high levels, A3H HapI can also elicit potent anti-viral activity *in vitro* and is resistant to Vif degradation [20]. A recent study reported a negative association between genotype HapII and A3F/H DNA editing signature on HIV-1 DNA sequences from early infected patients, suggesting that Vif-resistant haplotypes could contribute to HIV-1 diversification *in vivo* [46]. To investigate if A3H HapI is efficiently packaged into virions compared to A3H HapII, we co-transfected 293T cells with pNL4–3ΔVif along with various amounts of A3H vectors. Infectivity assays were performed using MAGI cells to measure anti-viral activities of A3H HapI and HapII. As shown in Figure 4A and B, A3H HapII had strong anti-viral activity and was efficiently packaged into the virus (Figure 4B, lanes 1, 2, and 3). When expressed at a high level, A3H HapI also suppressed virus replication up to about 60%. Interestingly, even when expressed at a similar or higher level in comparison to A3H HapII (Figure 4B, compare lane 5 to lanes 3 and 4), HapI had lower anti-viral activities and the level of A3H HapI in the purified virus was significantly lower compared to A3H HapII.

### The low A3H HapI binding affinities to host RNAs are enhanced by the G105R mutation

Since RLYY(Y/F/H)W is also conserved in A3H HapI, we asked if A3H HapI was also associated with host RNAs, as was the case with A3H HapII. We performed immunoprecipitation of A3H HapI and HapII and examined a panel of HapII-associated RNAs, including Pol III RNAs 7SL, Y1, Y3, Y4, 5s RNA as well as GAPDH and β-actin mRNA. Because different amounts of proteins were pulled down, we measured the intensities of the bands and normalized them to A3H HapII. Interestingly, we saw little difference in the RNA binding for GAPDH, β-actin, and 5s RNA which are not specifically packaged into the HIV-1 virion (Figure 5A). By contrast, A3H HapI showed reduced binding affinities to Y RNAs and 7SL RNA, the latter which is found abundantly in the virus [34] (Figure 5A). Therefore, the reduced ability of A3H HapI to bind to 7SL RNA may explain its less efficient and accurate packaging into the virion compared to A3H HapII.

A3H HapI and HapII differ by only 3 amino acids: 105G/R, 121K/E/D and 178E/D. Previous reports demonstrated that amino acid 105G/R determines protein stability while 121 K/E/D determines A3H binding to Vif and its sensitivity to Vif-induced degradation [7], [14], [16], [18], [22], [23]. Interestingly, single mutation G105R was shown to not only restore the expression level of A3H HapI, but also increase its anti-viral activity and the ability to be packaged [14], [17], [22], [23]. Accordingly, we were interested in determining if G105R could also restore the ability of A3H HapI to interact with 7SL and Y RNAs. After transfecting 293T cells with empty vector, A3H HapI, HapII or HapI105R plasmids, the expressed A3H proteins in the cell lysates were subsequently immunoprecipitated and their association with RNAs compared. As expected, the single point mutation G105R in A3H HapI restored its ability to bind to 7SL and Y RNAs at levels similar to that of A3H HapII (Figure 5B).

### A3H HapIIΔ15N does not have anti-viral activity and cannot be packaged into the virus

A3H HapIII and HapIV both differ from HapII at position 15(Δ or N). Both A3H HapIII and HapIV were reported to have weak or no anti-viral activities, and A3H Δ15N has greatly reduced protein stability [22], [24], which results in the loss of anti-viral activity. However, whether A3H HapIIΔ15N can be packaged in virions and has anti-viral activity when expressed at a similar level to A3H HapII are unknown. To test this, we co-transfected 293T cells with pNL4-3Δvif and decreasing amounts of A3H HapII or HapIIΔ15N expression constructs. As expected, A3H HapII showed strong antiviral activity and could be efficiently incorporated into virions (Figure 6A). In contrast to A3H HapI, which was present in purified virions and had low levels of anti-viral activity, A3H HapIIΔ15N, even when expressed at high levels similar to A3H HapII, was not detectable in the viral lysate and did not display any anti-viral activity (Figure 6B, compare lanes 5 and 6 to lanes 2,3,4).

### A3H HapII Δ15N does not associate with cellular RNAs

We performed immunoprecipitation procedures as above to determine if A3H HapIIΔ15N could bind RNA, and the abilities of A3H HapIΔ15N and HapII to associate with cellular RNAs were compared using qRT-PCR. A3H variant protein band intensities were measured and normalized to A3H HapII. Surprisingly, The A3H Δ15N deletion not only significantly impaired the ability of A3H HapII to bind to 7SL RNA and abolished its ability to bind to Y RNAs, it also greatly reduced its associations with other RNAs such as GAPDH (Figure 7A).

The loss of anti-viral activity and viral packaging of A3H HapIIΔ15N observed was very similar to that of the mutation HapIIW115A in the RLYYHW motif. To understand the role of 15N in relation to RLYYHW RNA motif, homology modeling of the A3H HapII was carried out based on the crystal structures of both APOBEC2 and the carboxyl-terminal domain of A3G. The result predicted that N15 is close in space to the RLYYHW region (Figure 7B).

### RNA binding activities are correlated with the cellular localizations of A3H variants

Previous report from Ohainle, et al showed that subcellular localization of A3H has changed during primate evolution: while rhesus macaque A3H is mainly cytoplasm, chimpanzee A3H and human A3H HapI is mostly nucleus [16]. A recent study that followed up on the localization of human A3H revealed that in contrast to A3H HapI, HapII localizes predominantly in the cytoplasm [47]. Using HapI and HapII fusion proteins, the study also suggested that HapI enters the nucleus through passive diffusion while HapI is being actively retended in the cytoplasm [47]. However, the localization of other haplotypes of A3H are unknown. To examine whether A3H haplotypes vary in their subcellular localization, we transfected Hela cells with the C-terminally HA-tagged A3H HapI, HapII, HapIIΔ15N and HapII115A constructs. Forty-eight hours post-transfection, the cells were fixed and stained with a mouse anti-HA antibody followed by a secondary Texas Red anti-mouse antibody for examination by deconvolution microscopy. As shown in figure 8, consistent with previous results, 92% of A3H HapII localized predominantly in the cytoplasm while for A3H HapI, a range of different localization patterns were observed: in some cells (∼64%) it localized both in the nucleus and cytoplasm, while in others (∼36%) it was predominantly in the cytoplasm. Surprisingly, A3H HapIIΔ15N and HapII115A, which has lost RNA binding activity, were found to be localized predominantly in the nucleus. The red offset signals were increased for A3H HapI, HapIIΔ15N, and HapII115A which was expressed at lower levels compared to A3H HapII. Our results showed that subcellular localizations of the A3H variants were highly diverse, and the presence of A3H in the cytoplasm appeared to correlate with the RNA binding activity of this protein.

## Discussion

Up to seven haplotypes have been identified to circulate in the human population for the A3H gene [24], having different combinations of 4 SNPs (18R/L, 105R/G, 121K/D, 178E/D) and one codon deletion (15N/Δ). In the absence of the Vif protein, only A3H variants that carry the combination of amino acids 15N and 105R (A3H HapII, V and VII) demonstrate strong anti-HIV-1 activity. A3H HapI (105G) and other haplotypes that carry Δ15N were reported to have weak or no anti-viral activity [14], [16], [17], [18], [22], [23], [24]. Why A3H variants differ in their anti-viral capabilities is currently unclear. Here we show that the anti-viral activity of human A3H correlated with its ability to bind host RNA.

It is critical for APOBEC3 proteins to be incorporated into virion particles in order to elicit a strong anti-viral response [27], [35], [45]. 7SL RNA binding activity has been proposed to be directly associated with the strength of APOBEC3-mediated anti-viral suppression [35]. Consistent with previous findings [36], we found that the RNA binding motif RLYY(Y/F/H)W that is conserved on A3G and A3F and in all A3H haplotypes is required for their effective packaging into the virion. We observed similar RNA binding activity of A3H HapII as that found with A3G. Previous study suggests 115W to A mutation on this motif on HapII abolish A3H packaging [36]. We found that mutation of 115W not only impaired A3H HapII virion packaging but also abolished its RNA binding activity, arguing for the importance of RNA binding for virion packaging of A3H. This finding is also in agreement with a previous report that the NC domain of Gag is required for A3H HapII packaging [23].

A3H HapI, differing in only 3 amino acids from A3H HapII, was expressed at a lower level when introduced into 293T and HeLa cells and has reduced anti-viral activity [14], [16], [18], [22], [23], [24]. However, A3H HapI appears to have relatively greater anti-viral activity when expressed at a higher level [20]. In this study, we showed that even at similar expression levels, A3H HapI had significantly reduced anti-viral activity compared to A3H HapII and could not package into virions efficiently. Furthermore, although A3H HapI maintained similar RNA binding activity to non-specific RNAs such as GAPDH and β-actin, it associated with significantly less 7SL and Y RNAs compare to HapII. Previous studies have shown that the G105R mutation restores anti-viral activity of A3H HapI [14], [17], [23], [24]. Here we determined that introducing an R to G mutation at 105 restored the A3H HapI RNA binding activity. Interestingly, when expressed at a high level, A3H HapI was detected in the virion, indicating that it was packaged via other mechanisms, possibly mediated by the MA-CA region of Gag [23].

A3H HapIII differs from A3H HapII only at one amino acid (15Δ/N), while A3H HapIV also has a deletion at position 15 as well as an additional mutation (18L/R). It was reported that Δ15N destabilizes A3H [16], but it is not clear if A3H HapIII and HapIV possess any anti-viral activity at all. Here we were able to express A3H HapIIΔ15N at a similar level to that of HapII, and strikingly, this deletion completely abolished A3H packaging into the virion as well as anti-viral activity. Remarkably, A3H HapIIΔ15N also lost almost all RNA binding activity. Our homology modeling of A3H HapII based on the APOBEC1 and APOBEC3G C-terminal domain showed that 15N sits closely to the putative RLYYHW RNA binding motif. We hypothesize that 15N is either directly involved in RNA binding or at least is important for maintaining the structure of the RNA binding pocket of A3H.

Members of the APOBEC3 family have diverse anti-viral and anti-retrotransposition activities [1], [2], [48], [49], [50], [51]. Here we showed that similar to A3F and A3G, A3H HapII had strong anti-viral activities while A3H HapI, like A3C, had modest anti-viral activity. Meanwhile, A3H HapIII/IV, like A3A, exhibited no anti-viral activity. The potency of A3H anti-viral activity was directly correlated with its ability to associate with cellular RNAs such as 7SL RNA and its ability to efficiently package into HIV-1 viral particles. We have identified a putative RNA binding site RLYYHW for A3H HapII and showed that 105R and 15N are essential for RNA binding activity.

Besides having diverse anti-viral activities, APOBEC3 proteins also show distinct features in cellular localization [4], [23], [52], [53], [54]. A3H HapII localized predominantly in the cytoplasm, while A3H HapI was found in both the cytoplasm and nucleus and A3H HapIIΔ15N primarily in the nucleus. Strikingly, we found that the localization of A3H variants correlated with their RNA binding activities; that is, A3H proteins that had higher affinities to 7SL and Y RNAs localized in the cytoplasm. Indeed, A3H HapIIW115A which lost its RNA binding activity also localized to the nucleus. Interestingly, a previous report had identified A3G amino acids 113–128 that includes the RNA binding RLYYFW motif as a cytoplasmic retention signal [52]. The authors concluded that RNA binding is not important for cytoplasmic retention by showing that the N-terminus of A3G is not associated with radiolabeled RNA but is still localized in the cytoplasm [52]. However, more sensitive detection methods have been developed, and a recent study concluded that the N-terminal A3G domain is actually responsible for association with specific host RNAs such as 7SL RNA [35]. Moreover, Stenglein et al. also reported that F126S and W127A as critical residues determining A3G cytoplasmic localization [55]. Therefore, it is likely that RNA binding is involved in regulating cytoplasmic localization of A3G. In the case of A3H, we have observed an association of RNA binding and cytoplasmic localization, suggesting that RNA is also involved in regulating A3H localization. Previous study has reported that both HapI and HapII associate with similar molecular mass complex [47], we therefore speculate that RNA binding may either play a role in cytoplasmic retention or mask critical amino acids responsible for nuclear proteins to interact with A3H. However, the specific mechanism responsible for A3H cellular localization requires further study.

It is striking that natural amino acid changes within A3H can give rise to variants that are highly diverse in anti-viral activities and cellular localizations. Distinct cellular localizations suggest that A3H variants interact with different proteins and/or nucleic acids and may have distinct functions within the cell. Previous reports have demonstrated that A3H variants distribute differently in geographically distinct human populations. For example, A3H HapII, III and IV are most commonly found in African populations, while HapI is the most common haplotype among Asians and Europeans [7], [14], [16], [22], [23], [24]. Different selection pressures may have driven A3H into having distinct functions, and it will be intriguing to study the inhibitory activities of A3H variants against other viruses.
